# Sex- and Ethnicity-related Differences in Pheochromocytoma/Paraganglioma

**DOI:** 10.1210/jendso/bvae070

**Published:** 2024-04-09

**Authors:** Peter Istvan Turai, Peter Igaz

**Affiliations:** Department of Internal Medicine and Oncology, Faculty of Medicine, Semmelweis University, 1083 Budapest, Hungary; Department of Internal Medicine and Oncology, Faculty of Medicine, Semmelweis University, 1083 Budapest, Hungary; Department of Endocrinology, Faculty of Medicine, Semmelweis University, 1083 Budapest, Hungary

**Keywords:** pheochromocytoma, paraganglioma, genetics, sex, ethnicity

Pheochromocytoma/paraganglioma (PPGL) is unique for having the highest rate of heritability among human tumors. A total of 40% to 50% of PPGL are caused by germline pathogenic variants (PV) in a set of approximately susceptibility 20 genes [1, 2]. Two major pathomechanisms were established (ie, the pseudohypoxia pathway [cluster 1 including clusters 1A and 1B] and kinase signaling [mTOR/RAS, cluster 2]), and recently a third pathway involving Wnt signaling has also been identified [3].

In their recent manuscript, Richter and Bechmann from the University of Dresden (Germany) present their findings in a retrospective analysis, where they compared the distribution of PV and reported clinical features of PPGL in European and Asian populations and related to sex [4]. Several molecular differences have been described between males and females and among different populations in various tumors [5], and this study sheds light on the relevance of these in chromaffin tumors as well. These particularly intriguing perspectives further highlight the growing interest in personalized medicine. The authors made several interesting observations.

Regarding demographic distribution, the dataset included 2162 patients with PPGL and a female percentage of 51.1%. The age at diagnosis did not differ significantly between Asian and European populations. Subgroup analysis revealed that Asian patients were diagnosed significantly earlier than European patients with *SDHB*- and *RET-*associated tumors. Because *SDHB* is the major gene associated with metastatic PPGL [6], this finding is particularly interesting.

Concerning germline PVs and sexual distribution, germline PVs in cluster 1A and 1B genes were more abundant in males, whereas cluster 2 genes showed a higher prevalence in females. *RET* and *TMEM127* gene alterations were associated with female, whereas *SDHB* and *VHL* with male sex. Somatic *EPAS1* PVs were significantly more common in females both in European and Asian populations, and *FGFR1* PVs were more common in males. Somatic *EPAS1* PVs were more common in PPGL detected in patients with congenital cyanotic heart disease [7]. As pointed out by the authors, the sex-related difference in the prevalence of somatic *EPAS1* PVs might be related to differences in blood and cardiovascular parameters and response to hypoxia between males and females [4]. However, this can only be considered pure hypothesis at present.

Regarding the prevalence of driver genes and demographic distribution, germline PVs in cluster 1A genes were more abundant among European patients, whereas cluster 1B and cluster 2 genes showed higher prevalence in Asian patients. The clinical presentation in males and females also showed differences: males presented with more abdominal or thoracic paraganglioma (35.6% vs 28.4%) and metastases (20.3% vs 15.3%). The increased prevalence of metastases in males might be related to a slightly worse prognosis. Females were more often diagnosed with head and neck paraganglioma. Europeans had proportionally more head and neck paragangliomas, whereas Asians had more paraganglioma and pheochromocytoma.

Although the differences between the 2 sexes and regions are not huge, these could nevertheless represent relevant differences.

In future research, exploring the correlation between sexual and demographic distribution with the recently identified third cluster involving Wnt signaling [3] would also be interesting. The differential genetic composition might affect response to treatment in different populations. Gaining insight into these molecular subgroups across various demographic regions may enhance the efficient regional distribution of potential advanced, targeted therapeutic drugs used for PPGL as a rare disease in the future.

A limitation of the study is certainly related to the lack of precise ancestry data in the studies reported. The authors excluded findings on populations from countries of mixed ancestry (eg, United States, Israel), but even so, the populations included cannot be regarded as homogeneous, especially in Europe. Because PPGL is associated with considerable heritability, ancestral differences could be relevant. To overcome this problem, the use of ancestry-informative markers (AIMs) would be an option in future studies. In the field of population genetics, AIMs refer to specific sets of single-nucleotide polymorphisms distinguished by markedly varying frequencies among distinct populations [5]. These markers can be used for 2 main purposes: first, to identify individuals who belong to a particular population group, and second, to address population stratification resulting from differences in population frequencies between cases and controls. For future investigations, it would be intriguing to see the AIM-matched PVs in patients with PPGL across diverse populations to further define population heterogeneity, which may also correspond to a genetic heterogeneity. Polymerase chain reaction-based assays could be effectively used for the selected AIMs as a straightforward and cost-efficient method to account for differences in continental ancestry in ethnically diverse populations [8].

Enhanced comprehension of the distribution and variations in clinical presentation among Asian and European and male-female populations with PPGL could lead to improved understanding of PPGL pathogenesis. This could even affect screening in specific at-risk groups. This accomplishment holds significant relevance from both patient and public health standpoints because early PPGL diagnosis can lead to an effective cure and prevention of severe, potentially life-threatening complications. Given the rarity of PPGL, conducting cost-effectiveness studies is also crucial to ascertain which groups of patients would derive the greatest benefit from targeted drug therapeutic trials.

## Disclosure

The authors have no competing interests to report.

## Abbreviations

AIM: ancestry-informative marker
PV: pathogenic variant
PPGL: pheochromocytoma/paraganglioma
